# Linkage of living microbial biomass, function, and necromass to soil organic carbon storage along a chronosequence of *Larix principis-rupprechtii* plantation in North China

**DOI:** 10.3389/fmicb.2025.1588030

**Published:** 2025-05-26

**Authors:** Mengyun Yang, Xiaomeng He, Jingfei Liang, Qiang Liu, Lihua Fu, Xiaodong Cui, Shaohui Huang, Haoan Luan

**Affiliations:** ^1^College of Forestry, Hebei Agricultural University, Baoding, China; ^2^Saihanba Mechanical Forest Farm, Chengde, China; ^3^Hebei Fertilizer Technology Innovation Centre, Institute of Agricultural Resources and Environment, Hebei Academy of Agriculture and Forestry Sciences, Shijiazhuang, China

**Keywords:** stand age, *Larix principis-rupprechtii*, soil aggregates, microbial characteristics, soil organic C dynamics

## Abstract

**Introduction:**

Clarifying the temporal dynamics of soil organic carbon (SOC) characteristics within aggregates and its underlying microbially mediated mechanisms is essential for long-term SOC sequestration in forest ecosystems; nevertheless, this information remains largely unknown during stand development.

**Methods:**

Five *Larix principis-rupprechtii* plantations of different ages (7a, 18a, 25a, 34a, and 44a) at the Saihanba Mechanical Forest Farm were chosen to elucidate the temporal variations in SOC characteristics and microbial attributes within aggregates (>2 mm, 2−0.25 mm, and <0.25 mm) following reforestation, based on ^13^C NMR, phospholipid fatty acid (PLFAs) analysis, micro-plate enzyme technique, and amino sugar analysis, etc.

**Results:**

Results demonstrated that as stand ages increased, aggregate stability as well as aggregate-associated SOC, microbial residue C (MRC), hydrolytic exo-enzymatic activities, and microbial biomass (as indicated by total PLFAs) initially increased and subsequently decreased, with most parameters peaking in the 18a stand, which indicated that long-term *Larix principis-rupprechtii* plantations (>25a) were not favorable for promoting microbial growth, hydrolytic functions, and microbial metabolism. Besides, regardless of the stand age, the above-mentioned indices were generally higher in larger aggregates (>2 mm and 2−0.25 mm) compared to smaller aggregates (<0.25 mm). Notably, the increased stand ages (i.e., 34a and 44a) or decreased aggregate sizes (<0.25 mm) enhanced SOC stability (as indicated by the recalcitrance index) and oxidative exo-enzymatic activities, as well as enlarged MRC (especially fungal residue C) contribution to SOC. The partial least squares path model highlighted that SOC stocks were primarily regulated by MRC, while the microbial community altered SOC stability by modulating exo-enzyme activities.

**Discussion:**

These results offered novel insights into elucidating the coupling connections between microbial attributes and SOC sequestration during forest development in northern China.

## 1 Introduction

Soil organic C (SOC), the biggest terrestrial C reservoirs (ca. 2,344 Gt) (Sakschewski et al., 2016), is receiving increasing attention due to its potential role in alleviating global warming and maintaining terrestrial ecological stability (Stockmann et al., 2013; Liu Q. et al., 2024). Nevertheless, since the Industrial Revolution, a series of human activities, such as extensive deforestation, urbanization, and overgrazing, have resulted in large amounts of CO_2_ emissions and SOC loss (Wiesmeier et al., 2019). Afforestation (i.e., planted forests), an effective approach to mitigate global CO_2_ emission and promote SOC stocks, has been developed rapidly in recent decades (Liang et al., 2022). By 2023, the global area of planted forests had reached 360.22 million ha, accounting for ca. 7.35% of the world’s total forest area (Xu et al., 2024). Consequently, it is imperative to assess soil C sequestration capability of these newly established planted forests and to elucidate the underlying mechanisms influencing this capacity, in order to deeply evaluate the role of afforestation in mitigating global climate change (Wang et al., 2024).

Recent research revealed that the forest’s capability for SOC sequestration is associated with stand age, as the variations in forest structure and understory vegetation with stand age can affect soil microenvironment and litter input conditions (Mujuru et al., 2014; Dong et al., 2020). Nevertheless, studies examining changes in SOC stocks across stands of varying ages had yielded conflicting results, reporting positive (Abaker et al., 2016), negative (Liu et al., 2023), and no effects (Jing et al., 2022) on SOC storage. These inconsistent results are likely attributed to the variations in forest species, climate, and edaphic characteristics (Chen et al., 2020; Wang et al., 2024). To better elucidate SOC dynamics, it is essential to address this knowledge gap regarding the underlying mechanisms for SOC sequestration along stand development.

Researchers revealed that the processes involved SOC cycling, such as degradation and polymerization, etc., are primarily regulated by microbes and their secreted exo-enzymes (Shao et al., 2018; Sokol et al., 2022). Recently, the response of stand ages to microbial attributes has been widely attracting scholarly attention (Wang et al., 2021; Zhang et al., 2023). Studies have demonstrated that stand age, a crucial parameter for assessing the rewilding level of plantations post-afforestation, can strongly influence the dynamics of microbial communities and exo-enzymatic activities through the modulation of understory vegetation composition and the exogenous resources (e.g., litter debris and root depositions) inputs (Zhang et al., 2023; Liu X. et al., 2024). Notably, traditional perspectives emphasize that SOC is mainly derived from plant-originated C, with microbes accounting for <4% of SOC and playing a crucial role in SOC decomposition and mineralization (Wang et al., 2017). Nevertheless, increasing evidence suggests that microbial anabolic functions are important in mediating persistent soil C storage (Shao et al., 2018; Hu H. et al., 2024). Based on the “microbial C pump” theory, Liang et al. (2017) indicated that microbial residues (i.e., microbial-derived C), producted through microbial anabolism, are considered to be the important constituents of stable SOC pools and contribute approximately 50–80% to SOC storage. Taken together, the study of combining microbial communities, exo-enzymes, and microbial residues, can comprehensively provide several valuable insights for elucidating microbial-driven SOC dynamics with plantation development (Ma et al., 2023). Yet, to date, the integrated study of the above-mentioned microbial attributes during the development of plantations is lacking.

To further elucidate the temporal variations in the aforementioned microbial attributes during stand development, they should be investigated at different spatial scales (e.g., the aggregate scale) (Chen et al., 2014; Mao et al., 2023). Soil aggregates, which serve as the basic units of soil structure, are the primary “sites” for SOC storage and act as “hotspots” for microbial activity (Gupta and Germida, 2015; Kuzyakov and Blagodatskaya, 2015). Recent studies have identified distinct distribution patterns of microbial attributes within different aggregates, attributed to their spatial heterogeneity and diverse physicochemical characteristics (e.g., pore properties and nutritional conditions) (Yao Y. et al., 2019; Wang and Hu, 2023). However, the impacts of aggregate size on microbial attributes along stand development remains inconsistent, with studies reporting increases, decreases, or no changes (Liao et al., 2023; Mao et al., 2023). To address this knowledge gap, an integrated study of microbial attributes, i.e., microbial communities, exo-enzymes, and microbial residue dynamics, within aggregates, to some extent, will enhance our understanding of microbial-driven soil C dynamics during forest development.

To fulfill our research objectives, five distinct age classes of *Larix principis-rupprechtii* plantations, i.e., 7a, 18a, 25a, 34a, and 44a, were selected from the Saihanba Mechanized Forest Farm, which represents the biggest plantation base in China (Wang Z. et al., 2018). It is noteworthy that since 1962, afforestation efforts, primarily involving *Larix principis-rupprechtii*, have been systematically and continuously implemented in Saihanba to address the issue of land desertification resulting from previous indiscriminate logging practices (Zhang et al., 2022). Consequently, *Larix principis-rupprechtii* of various ages are prevalent throughout this region. In this study, based on a series of technologies (e.g., ^13^C NMR, phospholipid fatty acids, exo-enzyme, and microbial residue analysis, etc.), we attempt to clarify the temporal changes in SOC dynamics within aggregates and the underlying microbial-driven mechanisms resulting in these variations after afforestation. Specifically, we hypothesized that (i) the growth and metabolism of microbes would be progressively strengthen during the development of plantations due to increased nutrient availability from annual continuous litter inputs; (ii) the variations in aggregate-related nutrient and pore properties induced by stand ages would alter the contents of microbial biomass and residues within soil aggregates; and (iii) the storage and composition of SOC would change along stand development, which were closely linked to the temporal changes in microbial attributes (i.e., microbial community, exo-enzymes, and microbial residues). These results can offer several valuable information for the sustainable development of planted forests in northern China.

## 2 Materials and methods

### 2.1 Study sites description and experimental design

**Figure 1:**
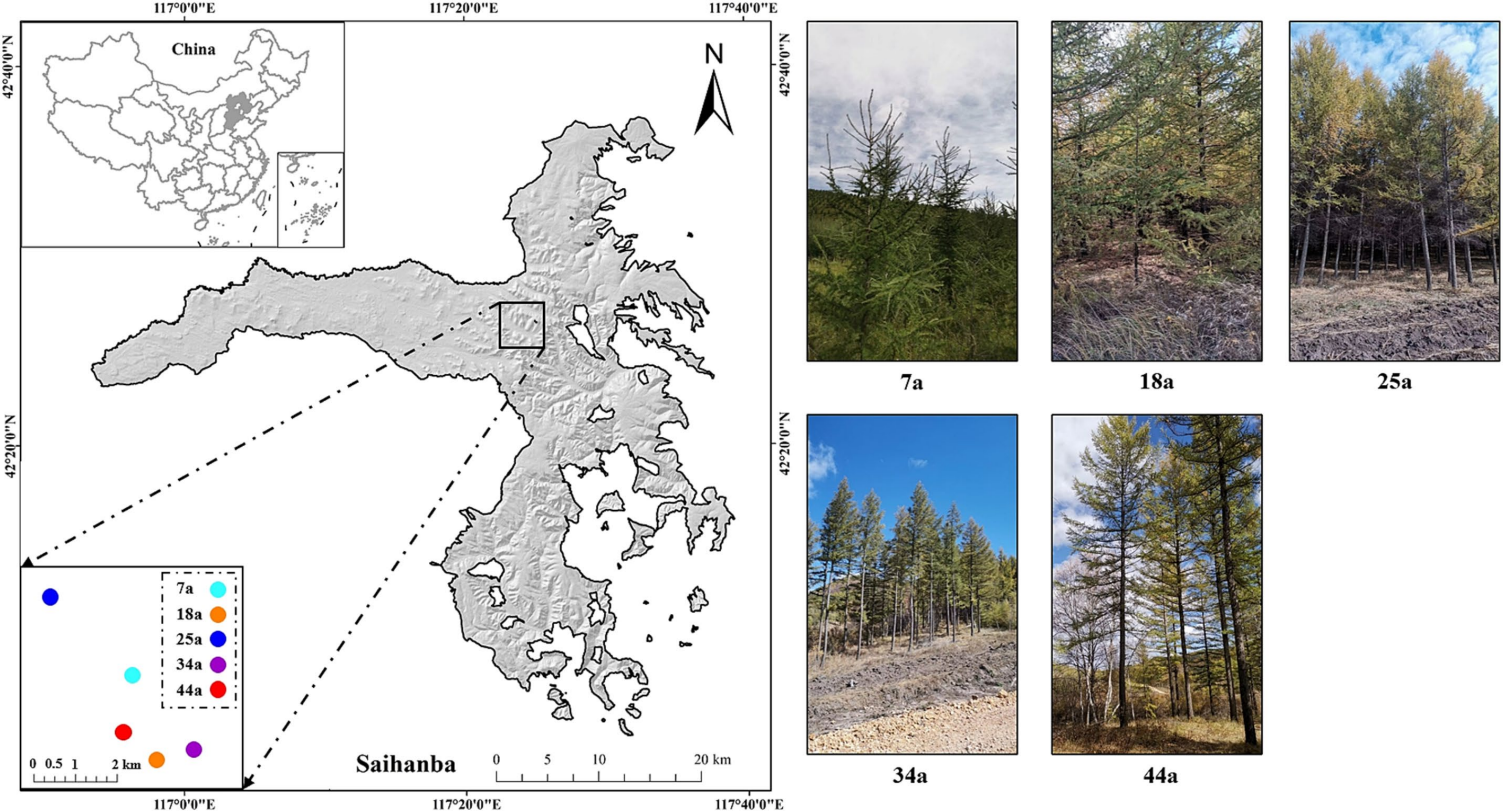
The study area and layout of plots for the five different-aged *Larix principis-rupprechtii* plantations.

The study was conducted at the Saihanba Mechanical Forest Farm, located in Hebei Province, North China (42°10′–42°50′N, 117°12′–117°30′E) with an elevation between 1,100 and 1940 m (Figure 1). This region belongs to a typical forest-steppe eco-tone with a temperate continental monsoon climate. The mean annual temperature and precipitation are −1.4°C and 450.1 mm, respectively. The average annual frost-free period is 68 days. The soils in the study site belong to brown soil (FAO, 2014). Besides, The cold-temperate coniferous forests, e.g., *Larix principis-rupprechtii* (shorted as larch), are the major forest types in this region due to their extreme cold tolerance and strong adaptability to soil.

The means of space-for-time substitution, a valuable method to survey soil temporal changes, was suitable for evaluating the temporal dynamics of forest’s soil evolution (Sparling et al., 2003). In the present study, five different-aged larch plantations, i.e., young (7a), middle-aged (18a and 25a), near mature (34a), and mature (41a) forests (see Figure 1), were selected to elucidate the temporal changes of soil characteristics that occur during the growth and development process of larch plantation. Three plots (20 m × 30 m) were randomly selected for investigation and sampling from each age of larch plantation. Besides, to be regarded as independent replications, each plot of the same stand age is separated by at least 0.2 km; meanwhile, all plots are within 10 km to ensure similar climatic and edaphic conditions. Other detailed information was shown in Table 1.

**Table 1:** The basic information, as well as the distribution characteristics of soil aggregates in the selected five aged stands of *Larix principis-rupprechtii* in the study sites.

Characteristics	Stand age	7a	18a	25a	34a	44a
Basic information	Elevation (m)	1,665	1,668	1,617	1,675	1,661
	Slope aspect	Northeast	East	North	East	Northeast
	Slope gradient (°)	8	4	10	11	6
	Mean tree height (m)	4.9	8.8	11.9	15.9	19.5
	Mean DBH (cm)	5.4	9.7	13.7	18.6	22.5
	Stand density (trees ha^−1^)	3,143	2,449	2,539	1,447	1,278
Aggregate distribution (%)	>2 mm	41.2 ± 2.5b	50.5 ± 0.7a	47.8 ± 4.2a	35.2 ± 0.5c	30.8 ± 1.4d
	0.25–2 mm	42.5 ± 1.4a	34.3 ± 3.2b	35.0 ± 3.3b	42.0 ± 3.4a	41.0 ± 2.4a
	<0.25 mm	16.3 ± 3.8c	15.1 ± 2.7c	17.1 ± 1.4c	22.8 ± 3.4b	28.2 ± 1.0a
Aggregate stability	MWD (mm)	2.56 ± 0.13b	2.93 ± 0.02a	2.81 ± 0.17a	2.26 ± 0.04c	2.04 ± 0.05d
	GMD (mm)	1.46 ± 0.18b	1.72 ± 0.09a	1.58 ± 0.13ab	1.15 ± 0.09c	0.96 ± 0.01c

The aggregate-associated data was shown as means values ± S.E. (*n* = 3). Different lower-case letters indicate significant differences (*p* < 0.05) among differently-aged *Larix principis-rupprechtii* stands. DBH, diameter at breast height. MWD, mean weight diameter; GMD, geometric mean diameter.

### 2.2 Soil sampling and aggregate fractionation

In late August 2023, five undisturbed soil samples were randomly collected from the surface soil (0–20 cm depth) under the canopy of the larch stand in each plot and placed in a hard plastic box (20 cm × 12 cm × 6 cm) to ensure that they could sustain their original structure during transport to the laboratory. In total, 15 soil samples (3 replicates × 5 stand age) were stored in 4°C conditions and immediately transferred to the laboratory for further analysis. Then, after the removal of impurities (e.g., leaf debris and stone, etc.), these soil samples were gently broken along natural fracture planes and passed through an 8 mm sieve for determination of aggregate fractionation.

Based on the optimal-moisture sieving method, the above soil samples were manually fractionated through a nest of 2 sieves (2 mm and 0.25 mm) into three aggregate-size classes: >2 mm, 2–0.25 mm, and <0.25 mm aggregates. The specific procedure was described in Mao et al. (2021). Then, the aggregate samples were separated into two parts. One part was air-dried and passed through 0.15 mm sieve to determine aggregate-associated physicochemical properties and microbial residues; the other part was stored at −80°C for the determination of aggregate-associated microbial characteristics.

### 2.3 Determination of soil physicochemical properties

Soil pH was measured with a glass electrode at a water-to-soil ratio of 2.5:1 (v/w). Soil organic C (SOC, i.e., total C, due to soils being acidic) and total nitrogen (TN) were measured by the dry combustion method using an elemental analyzer (VELP EMA 502, Italy) (Vitti et al., 2016).

### 2.4 Soil microbial community (PLFAs) analysis

The aggregate-associated microbial community was classified and quantified by the phospholipid fatty acids (PLFAs) analysis, as described by Kramer et al. (2013). Briefly, the frozen-dried soil samples were treated with mild alkaline methanolysis to form fatty acid methyl esters (FAMEs), and then dissolved in hexane and estimated by gas chromatography (Agilent 6,890 GC, Santa Clara, CA, USA) with the MIDI peak identification 4.5 software (MIDI Inc., Newark, DE). Subsequently, 24 individual PLFAs were chosen and quantified based on the internal standard (C19:0), and classified into fungi, bacteria, and actinomycetes, etc. (Supplementary Table S1). The contents of PLFAs were expressed in units of nmol g^−1^.

### 2.5 Soil exo-enzyme activity analysis

Seven exo-enzyme activities (five hydrolytic exo-enzymes and two oxidative exo-enzymes; Supplementary Table S2) were determined to evaluate microbial functional attributes using the micro-plate enzyme method (Zhang et al., 2015). Five labeled fluorogenic substrates and *L*-DOPA substrate (Supplementary Table S2) were applied for the determination of hydrolytic and oxidative exo-enzymatic activities, respectively. The detailed procedure was referred to Luan et al. (2020). Then, the hydrolytic or oxidative exo-enzymatic activities were measured through a microplate reader (Synergy 2, Biotek, USA) with 365 nm excitation and 450 nm emission filters or 450 nm absorbance, respectively. The seven exo-enzyme activities were calculated as nmol g^−1^ soil h^−1^.

### 2.6 Soil microbial residue (amino sugar) analysis

The amino sugars (AS), valuable indicators widely used to describe microbial necromass, were quantified by the procedure described by Ding et al. (2010). The AS generally contains three constitutes [i.e., glucosamine (GluN), galactosamine (GalN), and muramic acid (MurA)], which were extracted (i.e., hydrolysis, filtration, nitrogen-evaporation) and derivatized into aldononitrile derivatives. Finally, they were determined by the internal standard myo-inositol, which was added before hydrolyzation. The sum of GluN, GalN, and MurA was used to evaluate the total microbial necromass pool.

### 2.7 Solid-state ^13^C NMR analysis

The SOC chemical composition was assessed by solid-state ^13^C NMR spectroscopy. Before NMR measurements, aggregate samples were repeatedly pre-treated using hydrofluoric acid (5%) to eliminate paramagnetic compounds (i.e., Fe^3+^ and Mn^2+^, etc.) following Zhuang et al. (2011). The ^13^C NMR spectra were obtained on a Bruker AVANCE 400 NMR spectrometer (Germany) equipped with a 4-mm probe. Besides, the spectra were divided into four major regions, i.e., alkyl C (0–45 ppm), O-alkyl C (45–110 ppm), aromatic C (110–160 ppm), and carbonyl C (160–190 ppm) (Simpson and Simpson, 2012), and calculated by integration with the MestReNova 8.0 software (Mestrelab Research, Santiago de Compostela, Spain).

### 2.8 Calculations

#### 2.8.1 The indices of soil aggregate stability

By using the mass proportions of >2 mm, 2–0.25 mm, and <0.25 mm aggregates, mean weight diameter (MWD; mm) and geometric mean diameter (GMD; mm) were calculated to evaluate the stability of soil aggregates (Wang S. et al., 2018):


$$\mathrm{MWD}=\sum_{i=1}^{3}(\mathrm{Xi}\times \mathrm{Wi})$$

$$\mathrm{GMD}=\mathrm{EXP}\sum_{i=1}^{3}(\mathrm{Wi}\times \ln(\mathrm{Xi}))$$

Where Xi is the average diameter of aggregates (mm); and Wi is the mass proportions of aggregates (%).

#### 2.8.2 The geometric mean of the exo-enzyme activities

The geometric mean of the assayed hydrolytic and oxidative eco-enzymatic activities (GH and GOR) were calculated as follows:


$$\mathrm{GH}=\sqrt[{5}]{\mathrm{\alpha G}\times \mathrm{\beta G}\times \mathrm{CBH}\times \mathrm{XYL}\times \mathrm{NAG}}$$

$$\mathrm{GOR}=\sqrt[{2}]{\text{PHOs}\times \text{Perx}}$$

Where the full names of αG, βG, CBH, XYL, NAG, PHOs, and PerX are shown in Supplementary Table S2.

#### 2.8.3 Microbial residue C

Fungal and bacterial residue C (FRC and BRC, g kg^−1^) were calculated as follows (Cheng et al., 2024):


$$\mathrm{FRC}=\frac{(\text{GluN}/179.2–2\times \text{MurA}/251)\times 179.2\times 9}{1000}$$

$$\mathrm{BRC}=\frac{\text{MurA}\times 45}{1000}$$

Where 179.2 and 251 are the molecular masses of GluN and MurA, respectively, and 9 is the conversion factor of fungal GluN to fungal C.

#### 2.8.4 The indices of SOC stability

Three indices of SOC stability were calculated based on the relative abundance of SOC functional groups (Guo et al., 2019):


$$\mathrm{AI}=\frac{\text{aromatic}\;C}{\text{alkyl}\;C+O-\text{alkyl}\;C+\text{aromatic}\;C}\times 100$$

$$\;\mathrm{RI}=\frac{\text{aromatic}\;C+\text{alkyl}\;C}{O-\text{alkyl}\;C+\text{carbonyl}\;C}$$

$$A/\mathrm{OA}=\frac{\text{alkyl}\;C}{O-\text{alkyl}\;C}$$

Where AI and RI are aromaticity index and recalcitrance index, respectively.

### 2.9 Statistical analysis

One-way analysis of variance (ANOVA) with Duncan tests was conducted to assess the effect of different stand ages (or soil aggregates) on the physicochemical and microbial variables. Two-way ANOVA based on 45 soil samples (5 stand ages × 3 soil aggregates × 3 replicates) was applied to compare the differences in these above-mentioned variables with the five stand ages and three soil aggregates as the main factors. These statistical analyses were conducted with SPSS 16.0 software (SPSS Inc. Chicago, IL, USA). By using CANOCO 4.5 software, principal component analysis (PCA) was applied to identify the differences of microbial community across five different-aged larch plantations within soil aggregates. Besides, the effects of microbial characteristics (i.e., microbial communities, exo-enzyme activities, and microbial residues) on SOC dynamics were explored by the partial least squares path model (PLS-PM; 1,000 bootstraps) with the packages “plspm” through the R software (version 3.6.1).

## 3 Results

### 3.1 Soil aggregates distribution and stability

The aggregates in the surface soils (0–20 cm) across five different-aged larch plantations were found to be dominated by >2 mm and 2–0.25 mm aggregates (30.8–50.5 and 34.3–42.5%, respectively), followed by the <0.25 mm aggregates (15.1–28.2%) (Table 1). The proportion of >2 mm aggregates were significantly (*p* < 0.05) higher by 16.0–64.0% in the 18a and 25a stands than those in other stands (7a, 34, and 44a). In contrast, the 18a and 25a stands contained lower proportions of 2–0.25 mm and <0.25 mm aggregates than other stands. Additionally, the values of MWD and GMD first increased and then decreased with increasing stand age, reaching the highest value at 18a stand (2.93 mm and 1.72 mm) (Table 1).

### 3.2 The physicochemical properties within aggregates

The physicochemical properties, e.g., soil organic C (SOC), total N (TN), SOC/TN, and pH within soil aggregates, are shown in Table 2. Overall, these physicochemical indices were significantly (*p* < 0.05) affected by stand ages (except for pH) and soil aggregates. The contents of SOC and TN as well as their ratios (SOC/TN) were observed highest in the 18a and 25a stands, followed by the 7a stand, and lowest in the 34a and 44a stands within aggregates; meanwhile, 0.25–2 mm aggregates contained higher values of SOC, TN, and SOC/TN by 15.2–26.8, 7.1–11.3, and 7.3–13.6%, respectively, than other aggregates (>2 mm and <0.25 mm). The soil pH was significantly (*p* < 0.05) higher in the 7a, 18a, and 25a stands than those in the 34 and 44a stands within aggregates; meanwhile, there were no significant differences in the values of pH across all aggregates (5.91–5.96) irrespective of the stand ages.

**Table 2:** The physicochemical properties within soil aggregates in differently-aged *Larix principis-rupprechtii* stands.

Indices	Stand age (S)	Soil aggregate size (A)			Effects		
		>2 mm	0.25–2 mm	<0.25 mm	A	S	A × S
SOC (g kg^−1^)	7a	26.9 ± 2.7b	30.7 ± 3b	24.1 ± 2.8ab	**	**	ns
	18a	33.2 ± 1.4a	40.0 ± 3.1a	30.7 ± 3.8a			
	25a	31.1 ± 2.2a	34.4 ± 2.3ab	26.8 ± 3.8a			
	34a	20.1 ± 1.8c	22.9 ± 4.4c	19.2 ± 3.5b			
	44a	20.1 ± 2.1c	23.4 ± 2.6c	18.6 ± 3.9b			
	Average	26.3B	30.3A	23.9B			
TN (g kg^−1^)	7a	2.45 ± 0.24a	2.56 ± 0.36ab	2.31 ± 0.3a	*	**	ns
	18a	2.58 ± 0.26a	2.88 ± 0.26a	2.50 ± 0.23a			
	25a	2.59 ± 0.28a	2.69 ± 0.34ab	2.35 ± 0.15a			
	34a	2.3 ± 0.12ab	2.42 ± 0.25ab	2.27 ± 0.43a			
	44a	2.06 ± 0.16b	2.27 ± 0.12b	2.09 ± 0.3a			
	Average	2.39AB	2.56A	2.30B			
SOC/TN	7a	11.0 ± 0.3bc	12.0 ± 0.6bc	10.4 ± 0.1b	**	**	ns
	18a	13.0 ± 1.3a	13.9 ± 0.8a	12.3 ± 1.1a			
	25a	12.1 ± 1.1ab	12.9 ± 1.1ab	11.3 ± 1.1ab			
	34a	8.8 ± 0.6d	9.4 ± 1.3d	8.5 ± 0.1c			
	44a	9.8 ± 0.4 cd	10.3 ± 0.6 cd	8.8 ± 0.5c			
	Average	10.9B	11.7A	10.3C			
pH	7a	6.27 ± 0.12a	6.24 ± 0.03a	6.23 ± 0.11a	ns	**	ns
	18a	6.16 ± 0.09a	6.13 ± 0.08a	6.12 ± 0.03ab			
	25a	6.14 ± 0.04a	6.09 ± 0.12a	6.10 ± 0.05b			
	34a	5.61 ± 0.08b	5.54 ± 0.10b	5.49 ± 0.08c			
	44a	5.61 ± 0.15b	5.53 ± 0.04b	5.63 ± 0.02c			
	Average	5.96A	5.91A	5.91A			

Data were shown as means values ± S.E. (*n* = 3 or 15). Different uppercase and lowercase letters indicate significant differences among different soil aggregates and differently-aged *Larix principis-rupprechtii* stands within the same soil aggregates at the *P* < 0.05 level, respectively. Two-way ANOVA are applied for identifying the effects of stand age (S), aggregate size (A), and their interaction (S × A) on the above-mentioned indices (The results are shown in the right side of the table). **, * and “ns” represent significant at *P* < 0.01, *p <* 0.05, and no significant differences (*P* > 0.05), respectively. SOC, soil organic C; TN, total N.

### 3.3 The microbial community composition within aggregates

**Figure 2:**
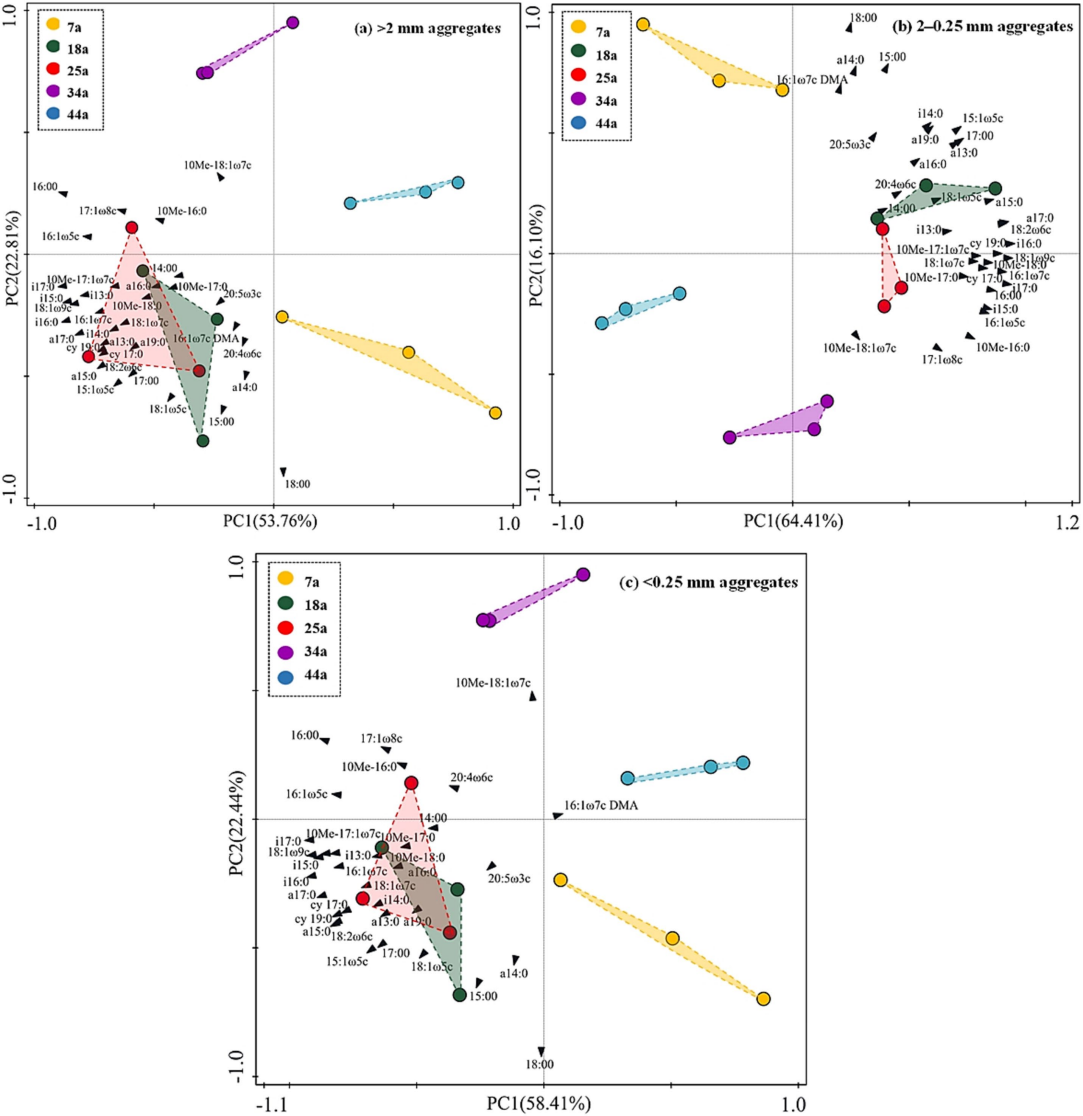
The principal component analysis (PCA) based on 24 individual PLFAs in the present study across differently-aged *Larix principis-rupprechtii* stands within different soil aggregates. The “10Me” in 10Me-18:1ω7c, 10Me-17:1ω7c, 10Me-16:0, 10Me-17:0, and 10Me-18:0 means “10-methyl”; the “i” in i13:0, i14:0, i15:0, i16:0, i17:0 means “iso”; the “a” in a14:0 a15:0, a16:0, a17:0 means “anteiso”; the “DMA” in 16:1ω7c DMA means “dimethyl acetal”; the “cy” in cy17:0 and cy19:0 means “cyclo”.

Table 3 revealed that the contents of total microbes (i.e., total PLFAs) showed a unimodal trend with increasing stand ages within aggregates. Specifically, the values of total PLFAs were found higher by 21.5–53.4, 23.0–72.2, and 25.0–72.2%, respectively, in the 18a and 25a stands than other stands within >2 mm, 2–0.25 mm and <0.25 mm aggregates; meanwhile, >2 mm and 2–0.25 mm aggregates owned higher contents of total microbes by 27.4% on average than <0.25 mm aggregates. The relative abundance of fungi was found significantly (*p* < 0.05) lower in the 7a stand than that in the 18a, 25a, 34a, and 44a stands within aggregates. Besides, the relative abundance of bacteria showed ranked in the order: 18a and 25a > 7a, 34a, and 44a within aggregates. Notably, regardless of the stand age, the 2–0.25 mm aggregates contained lower relative abundance of fungi and higher relative abundance of bacteria than the >2 mm and <0.25 mm aggregates. These results induced the higher F/B ratios to be found in the 34a and 44a stands (or the <0.25 mm aggregates) than the 7a, 18a, and 25a stands (or the >2 mm and 2–0.25 mm aggregates) (Table 3). The PCA results (Figure 2) further identified stand age could alter the microbial community structure within aggregates. Specifically, the microbial profiles within >2 mm, 2–0.25 mm, and <0.25 mm aggregates among different-aged stands owned similar and obvious boundaries, which were categorized into four groups, i.e., 7a vs. 18a and 25a vs. 34a vs. 44a.

**Table 3:** The contents of total PLFAs, the relative abundance of microbial subgroups as well as associated ratios (F/B) within soil aggregates in differently-aged *Larix principis-rupprechtii* stands.

Indices	Stand age (S)	Soil aggregate size (A)			Effects		
		>2 mm	0.25–2 mm	<0.25 mm	A	S	A × S
Total PLFAs (nmol g^−1^)	7a	27.1 ± 3.3b	32.6 ± 4.0b	22.9 ± 2.8b	**	**	ns
	18a	35.1 ± 1.5a	45.7 ± 2.8a	30.3 ± 1.4a			
	25a	37.3 ± 2.9a	41.2 ± 0.5a	31.0 ± 1.9a			
	34a	28.9 ± 2.7b	33.5 ± 3.4b	24.2 ± 2.3b			
	44a	24.3 ± 1.6b	26.5 ± 1.7c	20.4 ± 1.8b			
	Average	30.6B	35.9A	25.8C			
Fungi (%)	7a	8.5 ± 0.8b	7.6 ± 0.4b	9.5 ± 0.8b	**	**	ns
	18a	9.8 ± 0.4a	10.0 ± 0.2a	11.2 ± 0.3a			
	25a	10.7 ± 0.4a	9.6 ± 0.7a	11.9 ± 0.2a			
	34a	10.4 ± 0.1a	9.2 ± 0.1a	11.5 ± 0.1a			
	44a	10.2 ± 0.7a	9.6 ± 0.6a	12.0 ± 0.4a			
	Average	10.0B	9.2C	11.2A			
Bacteria (%)	7a	53.0 ± 1.1b	57.3 ± 2.3b	53.3 ± 1.3c	**	**	ns
	18a	58.0 ± 2.7a	62.0 ± 2.4a	58.1 ± 1.5a			
	25a	57.6 ± 3.8ab	61.1 ± 0.5a	57.4 ± 2.8ab			
	34a	53.3 ± 1.8b	57.3 ± 1.7b	53.3 ± 1.7c			
	44a	54.4 ± 1.7ab	58.6 ± 1.7ab	54.6 ± 1.4bc			
	Average	55.2B	59.3A	55.4B			
Actinomycetes (%)	7a	4.52 ± 0.71ab	3.78 ± 0.52b	4.29 ± 0.65ab	ns	*	ns
	18a	4.56 ± 0.07ab	4.04 ± 0.12ab	4.36 ± 0.07ab			
	25a	3.88 ± 0.99b	4.17 ± 0.22ab	3.88 ± 0.73b			
	34a	5.50 ± 1.23a	5.02 ± 0.98a	5.31 ± 1.15a			
	44a	4.99 ± 0.47ab	4.53 ± 0.41ab	4.76 ± 0.53ab			
	Average	4.69A	4.31A	4.52A			
F/B	7a	0.160 ± 0.012c	0.133 ± 0.002b	0.178 ± 0.01c	**	**	ns
	18a	0.169 ± 0.002bc	0.162 ± 0.006a	0.193 ± 0.002b			
	25a	0.185 ± 0.007ab	0.157 ± 0.012a	0.207 ± 0.006ab			
	34a	0.195 ± 0.004a	0.161 ± 0.004a	0.216 ± 0.005a			
	44a	0.194 ± 0.012a	0.163 ± 0.01a	0.220 ± 0.011a			
	Average	0.181B	0.155C	0.203A			

Data were shown as means values ± S.E. (*n* = 3 or 15). Different uppercase and lowercase letters indicate significant differences among different soil aggregates and differently-aged *Larix principis-rupprechtii* stands within the same soil aggregates at the *P* < 0.05 level, respectively. Two-way ANOVA are applied for identifying the effects of stand age (S), aggregate size (A), and their interaction (S × A) on the above-mentioned indices (The results are shown in the right side of the table). **, *, and “ns” represent significant at *P* < 0.01, *P <* 0.05, and no significant differences (*P* > 0.05), respectively. F, fungi; B, bacteria.

### 3.4 The exo-enzyme activities within aggregates

**Figure 3:**
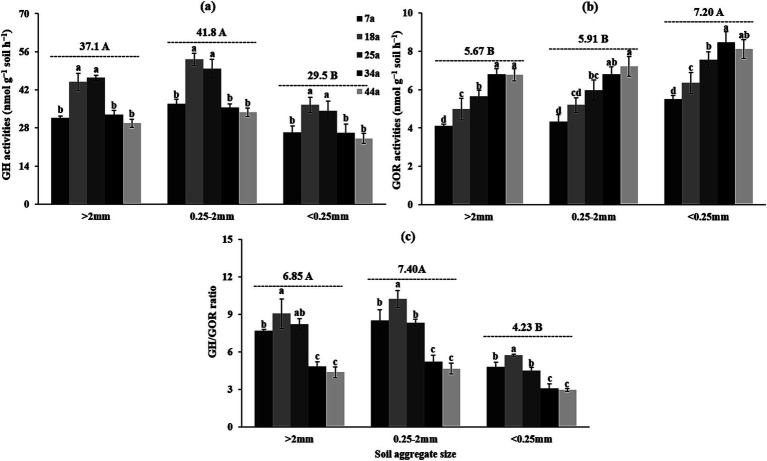
The geometric mean of the assayed exo-enzyme activities [**(a)** GH, **(b)** GOR and **(c)** GH/GOR] within soil aggregates across differently-aged *Larix principis-rupprechtii* stands. Different uppercase and lowercase letters indicate significant differences among different soil aggregates and differently-aged *Larix principis-rupprechtii* stands within the same soil aggregates at the *p* < 0.05 level, respectively. GH, the geometric mean of the hydrolytic exo-enzyme activities; GOR, the geometric mean of the oxidative exo-enzyme activities.

Table 4 and Figure 3 revealed that the activities of hydrolytic and oxidative exo-enzymes were significantly (*p* < 0.01) influenced by stand age and soil aggregates. For hydrolytic eco-enzymatic activities, these indices (e.g., GH, αG, βG, CBH, XYL, and NAG) within aggregates initially increased and subsequently decreased, with most parameters peaking in 18a or 25a stands. For oxidative exo-enzymatic activities, these indices (e.g., GOR, PHOs, and PerX) basically exhibited increasing trends as the stand age increased. Besides, regardless of the stand age, the >2 mm and 2–0.25 mm aggregates owned higher hydrolytic eco-enzymatic activities by 25.8–41.7% and lower oxidative exo-enzymatic activities by 21.8–26.7% than the <0.25 mm aggregates. Notably, the values of GH/GOR were consistently greater in the 18a stand (or >2 mm and 2–0.25 mm aggregates) than in the 7a, 25a, 34a, and 44a stands (or <0.25 mm aggregates) (Figure 3c).

**Table 4:** The exo-enzyme activities (nmol g^−1^ soil h^−1^) within soil aggregates in differently-aged *Larix principis-rupprechtii* stands.

Indices	Stand age (S)	Soil aggregate size (A)			Effects		
		>2 mm	0.25–2 mm	<0.25 mm	A	S	A × S
αG	7a	8.0 ± 0.3d	13.7 ± 2.2a	9.6 ± 1.7ab	**	**	ns
	18a	13.8 ± 0.4a	16.2 ± 0.5a	11.0 ± 2.0a			
	25a	12.7 ± 1.2ab	15.6 ± 1.4a	9.9 ± 0.6a			
	34a	10.8 ± 1.9bc	13.8 ± 1.3a	7.2 ± 1.1b			
	44a	10.1 ± 1.3 cd	13.5 ± 1.8a	8.5 ± 0.9ab			
	Average	11.0B	14.6A	9.3C			
βG	7a	74.8 ± 3.3b	87.9 ± 7.5b	67.8 ± 10.7b	**	**	ns
	18a	98.4 ± 7.3a	128.2 ± 19.2a	101.8 ± 14.5a			
	25a	103.5 ± 10.8a	115.9 ± 15.0a	69.8 ± 16.8b			
	34a	76.3 ± 10.1b	78.7 ± 10.4bc	62.6 ± 15.0b			
	44a	56.2 ± 4.8c	57.7 ± 6.1c	49.3 ± 8.3b			
	Average	81.8AB	93.7A	70.3B			
XYL	7a	42.7 ± 3.6b	40.0 ± 3.2c	25.6 ± 4.5b	**	**	*
	18a	63.1 ± 5.4a	66.3 ± 6.6a	34.5 ± 6.1ab			
	25a	68.0 ± 7.1a	54.6 ± 6.6b	42.6 ± 4.5a			
	34a	43.5 ± 6.7b	40.9 ± 4.6c	31.4 ± 2.3b			
	44a	46.9 ± 6.0b	42.2 ± 5.1c	29.4 ± 4.8b			
	Average	52.8A	48.8A	32.7B			
CBH	7a	47.2 ± 1.8bc	54.6 ± 9.6c	39.4 ± 8.5b	**	**	ns
	18a	71.0 ± 9.2a	90.0 ± 8.6a	66.6 ± 7.2a			
	25a	68.8 ± 1.9a	74.2 ± 10.5b	63.3 ± 10.1a			
	34a	53.5 ± 3.7b	54.7 ± 5.0c	44.7 ± 7.2b			
	44a	42.4 ± 3.1c	55.6 ± 3.4c	34.4 ± 7.2b			
	Average	56.6AB	65.8A	49.7B			
NAG	7a	27.0 ± 1.8b	26.5 ± 4.9bc	20.2 ± 0.8bc	**	**	ns
	18a	30.2 ± 2.2b	34.8 ± 4.4ab	25.9 ± 4.1a			
	25a	35.6 ± 4.2a	42.5 ± 5.2a	25.5 ± 4.1ab			
	34a	20.5 ± 2.8c	23.6 ± 3.4c	19.9 ± 2.7bc			
	44a	20.7 ± 2.1c	24.2 ± 4.5c	19.6 ± 1.3c			
	Average	26.8AB	30.3A	22.2B			
PHOs	7a	4.95 ± 0.31c	4.60 ± 0.47c	6.21 ± 0.55b	**	**	ns
	18a	4.72 ± 0.39c	4.68 ± 0.33c	5.88 ± 0.32b			
	25a	6.18 ± 0.20b	6.29 ± 0.29b	7.94 ± 0.91a			
	34a	7.07 ± 0.32a	7.19 ± 0.56a	8.15 ± 0.28a			
	44a	7.10 ± 0.09a	7.74 ± 0.66a	7.88 ± 0.50a			
	Average	6.00B	6.10B	7.21A			
PerX	7a	3.45 ± 0.36c	4.10 ± 0.25c	4.91 ± 0.72d	**	**	ns
	18a	5.29 ± 0.77b	5.79 ± 0.55b	6.87 ± 0.86c			
	25a	5.18 ± 0.51b	5.70 ± 0.74b	7.21 ± 0.14bc			
	34a	6.52 ± 0.30a	6.44 ± 0.32ab	8.78 ± 0.90a			
	44a	6.47 ± 0.62a	6.75 ± 0.40a	8.38 ± 0.55ab			
	Average	5.38B	5.76B	7.23A			

Data were shown as means values ± S.E. (*n* = 3 or 15). Different uppercase and lowercase letters indicate significant differences among different soil aggregates and differently-aged *Larix principis-rupprechtii* stands within the same soil aggregates at the *P* < 0.05 level, respectively. Two-way ANOVA are applied for identifying the effects of stand age (S), aggregate size (A), and their interaction (S × A) on the above-mentioned indices (The results are shown in the right side of the table). **, *, and “ns” represent significant at *P* < 0.01, *P <* 0.05, and no significant differences (*P* > 0.05), respectively. αG, α-Glucosidase; βG, β-Glucosidase; CBH, β-Cellobiosidase; XYL, β-Xylosidase; NAG, N-acetyl-glucosaminidase; PHOs, phenol oxidase; PerX, peroxidase.

### 3.5 The microbial residue C within aggregates

**Figure 4:**
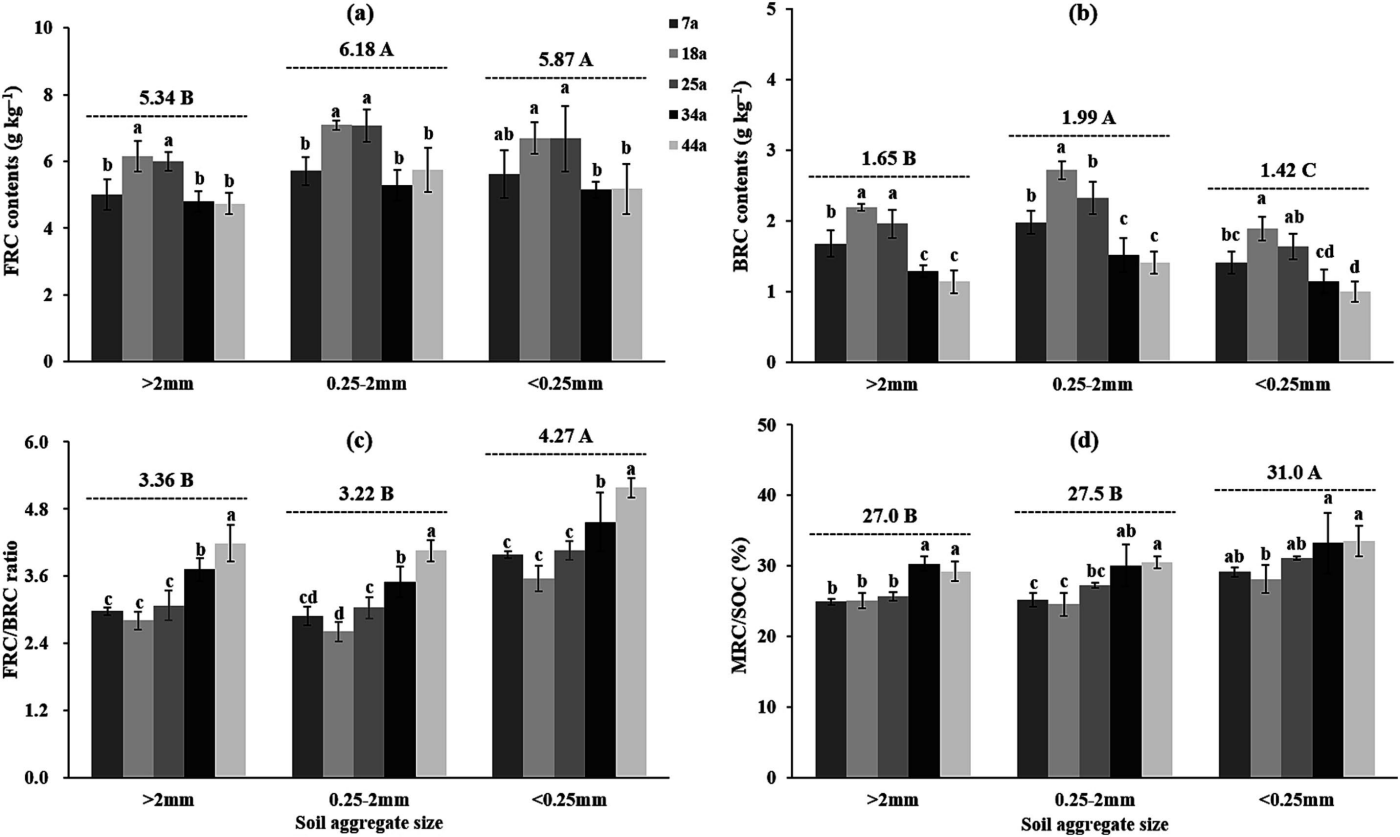
The contents of microbial residue C [**(a)** MRC, **(b)** FRC, and **(c)** BRC] as well as the associated ratio [**(d)** MRC/SOC] within soil aggregates across differently-aged *Larix principis-rupprechtii* stands. Different uppercase and lowercase letters indicate significant differences among different soil aggregates and differently-aged *Larix principis-rupprechtii* stands within the same soil aggregates at the *p* < 0.05 level, respectively. FRC, fungal residue C; BRC, bacterial residue C; MRC, microbial residue C; SOC, soil organic C.

The contents of amino sugars (total AS, GluN, GalN, and MurA; Table 5) and microbial residue C (FRC and BRC; Figure 4) basically and gradually increased from 7a to 14a stands but then declined in the 25a, 34a and 44a stands within aggregates; meanwhile, regardless of the stand age, the contents of FRC and BRC were higher by 5.3–15.6% and 20.6–40.9%, respectively, in the 2–0.25 mm aggregates than the >2 mm and <0.25 mm aggregates. Notably, the ratios of FRC/BRC and MRC/SOC were observed significantly (*p* < 0.05) higher in the 34a and 44a stands (3.50–5.18 and 29.2–33.5%) compared to the 7a, 14a, and 25a stands (2.61–4.06 and 24.6–31.1%) within aggregates; meanwhile, these indices were ranked as: [<0.25 mm aggregates (4.27 and 31.0%)] > [>2 mm and 2–0.25 mm aggregates (3.22–3.36 and 27.0–275%)].

**Table 5:** The amino sugars (mg kg^−1^) within soil aggregates in differently-aged *Larix principis-rupprechtii* stands.

Indices	Stand age (S)	Soil aggregate size (A)			Effects		
		>2 mm	0.25–2 mm	<0.25 mm	A	S	A × S
GluN	7a	610.1 ± 56.4b	697.7 ± 51.7b	668.6 ± 84.5ab	**	**	ns
	18a	753.6 ± 53.0a	873.6 ± 12.6a	804.8 ± 57.4a			
	25a	730.4 ± 35.3a	859.3 ± 59.7a	794.5 ± 114.2a			
	34a	574.5 ± 35.0b	634.6 ± 58.8b	608.6 ± 33.5b			
	44a	562.2 ± 40.7b	682.3 ± 78.1b	606.8 ± 87.4b			
	Average	646.1B	749.5A	696.6AB			
GalN	7a	372.8 ± 42.3 cd	432.7 ± 53.2b	386.4 ± 57.9bc	**	**	ns
	18a	482.8 ± 23.3a	589.3 ± 17.9a	488.9 ± 16.3a			
	25a	454.7 ± 49.8ab	511.0 ± 45.8ab	434.9 ± 50.2ab			
	34a	400.8 ± 30.9bc	462.6 ± 78.5ab	408.1 ± 67.9abc			
	44a	324.3 ± 26.4d	393.2 ± 104.9b	337.4 ± 6.5c			
	Average	407.1B	477.7A	411.1B			
MurA	7a	37.4 ± 4.1b	44.1 ± 3.5c	31.2 ± 3.6bc	**	**	ns
	18a	48.6 ± 1.1a	60.4 ± 3.0a	42.0 ± 3.7a			
	25a	43.6 ± 4.4a	51.8 ± 5.2b	36.5 ± 3.9ab			
	34a	28.7 ± 1.8c	33.8 ± 5.3d	25.3 ± 3.9 cd			
	44a	25.3 ± 3.6c	31.4 ± 3.6d	22.2 ± 3.1d			
	Average	36.7B	44.3A	31.5C			
Total AS	7a	1020.3 ± 51.9b	1174.5 ± 62.3b	1086.2 ± 138.1bc	**	**	ns
	18a	1285.0 ± 52.0a	1523.3 ± 6.8a	1335.7 ± 75.4a			
	25a	1228.7 ± 86.5a	1422.1 ± 103.8a	1265.8 ± 167.9ab			
	34a	1004.0 ± 64.7b	1131.0 ± 139.1b	1042.1 ± 103.9bc			
	44a	911.8 ± 30.3b	1106.9 ± 180.1b	966.4 ± 94.6c			
	Average	1090.0B	1271.6A	1139.3B			

Data were shown as means values ± S.E. (*n* = 3 or 15). Different uppercase and lowercase letters indicate significant differences among different soil aggregates and differently-aged *Larix principis-rupprechtii* stands within the same soil aggregates at the *P* < 0.05 level, respectively. Two-way ANOVA are applied for identifying the effects of stand age (S), aggregate size (A), and their interaction (S × A) on the above-mentioned indices (the results are shown in the right side of the table). ** and “ns” represent significant at *P* < 0.01 and no significant differences (*P* > 0.05), respectively. AS, amino sugar.

### 3.6 The SOC chemical composition within aggregates

The results of ^13^C NMR analysis (Table 6) demonstrated that the effects of stand ages and aggregate sizes on SOC functional groups were primarily focused on aromatic C (*p* < 0.01) and O-alkyl C (*p* < 0.01), rather than carbonyl C and alkyl C (*p* > 0.05). Specifically, the relative abundance of aromatic C within aggregates increased with stand ages (except for 34a in 0.25–2 mm aggregates), whereas the relative abundance of O-alkyl C showed an opposite trend (7a, 18a, and 25a > 34a and 44a). Regardless of the stand ages, the SOC in the >2 mm and 0.25–2 mm aggregates exhibited greater relative abundance of O-alkyl C and lower relative abundance of aromatic C than those in the <0.25 mm aggregates; meanwhile, no significant differences (*p* > 0.05) were observed in the relative abundance of carbonyl C and alkyl C across different aggregates (Table 6). Notably, the values of RI, AI, and A/OA within aggregates were all ranked as 44a > 18a, 25a, and 34a > 7a (except for the RI and A/OA within >2 mm aggregates); meanwhile, the SOC in <0.25 mm aggregates owned significantly higher values of RI, AI, and A/OA than those in >2 mm and 0.25–2 mm aggregates irrespective of the stand ages (Table 6).

**Table 6:** The relative abundance (%) of different SOC functional groups and their spectroscopic indices within soil aggregates in differently-aged *Larix principis-rupprechtii* stands.

Indices	Stand age (S)	Soil aggregate size (A)			Effects		
		>2 mm	0.25–2 mm	<0.25 mm	A	S	A × S
Carbonyl C	7a	5.22 ± 0.59b	5.94 ± 0.37a	6.93 ± 1.09a	ns	ns	*
	18a	6.38 ± 1.39ab	5.25 ± 1.24a	6.95 ± 0.74a			
	25a	6.21 ± 0.47ab	6.39 ± 0.92a	7.05 ± 0.36a			
	34a	6.89 ± 0.61a	6.54 ± 0.64a	5.09 ± 0.66b			
	44a	6.71 ± 0.39ab	6.33 ± 0.42a	6.18 ± 0.9ab			
	Average	6.28A	6.09A	6.44A			
Aromatic C	7a	12.2 ± 0.8c	12.9 ± 0.9b	14.0 ± 0.7c	**	**	ns
	18a	13.5 ± 0.5b	14.4 ± 1.2ab	14.7 ± 0.8bc			
	25a	15.4 ± 0.1a	15.2 ± 0.1a	16.3 ± 0.1ab			
	34a	15.4 ± 0.8a	14.3 ± 1.2ab	16.3 ± 1.4ab			
	44a	15.7 ± 0.2a	16.0 ± 0.9a	16.7 ± 1.4a			
	Average	14.5B	14.6B	15.6A			
O-alkyl C	7a	52.1 ± 0.8a	52.8 ± 0.4a	49.8 ± 1.3a	**	**	ns
	18a	52.4 ± 0.6a	50.9 ± 0.7ab	48.9 ± 1.9ab			
	25a	51.1 ± 1.7ab	50.1 ± 0.5bc	49.0 ± 0.2ab			
	34a	49.0 ± 2.6b	50.1 ± 2.5bc	48.6 ± 1.3ab			
	44a	49.0 ± 0.4b	48.0 ± 0.8c	47.3 ± 0.6b			
	Average	50.7A	50.4A	48.72B			
Alkyl C	7a	30.5 ± 1.2a	28.3 ± 0.2a	29.3 ± 0.9ab	ns	ns	ns
	18a	27.7 ± 2.0a	29.5 ± 2.2a	29.5 ± 1.0a			
	25a	27.3 ± 1.7a	28.3 ± 0.5a	27.7 ± 0.3b			
	34a	28.7 ± 2.2a	29.1 ± 1.9a	30.0 ± 0.7a			
	44a	28.5 ± 0.9a	29.6 ± 0.6a	29.8 ± 1.2a			
	Average	28.5A	29.0A	29.2A			
RI	7a	0.746 ± 0.040a	0.703 ± 0.021c	0.764 ± 0.010b	**	**	ns
	18a	0.702 ± 0.059a	0.781 ± 0.033ab	0.791 ± 0.043b			
	25a	0.746 ± 0.054a	0.770 ± 0.015bc	0.784 ± 0.005b			
	34a	0.793 ± 0.097a	0.768 ± 0.068bc	0.861 ± 0.024a			
	44a	0.794 ± 0.023a	0.842 ± 0.023a	0.870 ± 0.014a			
	Average	0.756B	0.773B	0.814A			
AI	7a	0.129 ± 0.009c	0.138 ± 0.009b	0.151 ± 0.006b	**	**	ns
	18a	0.144 ± 0.006b	0.152 ± 0.015ab	0.158 ± 0.010ab			
	25a	0.164 ± 0.001a	0.163 ± 0.002a	0.175 ± 0.002a			
	34a	0.166 ± 0.008a	0.153 ± 0.014ab	0.172 ± 0.016ab			
	44a	0.168 ± 0.003a	0.171 ± 0.010a	0.178 ± 0.015a			
	Average	0.154B	0.155B	0.167A			
A/OA	7a	0.585 ± 0.030a	0.537 ± 0.003b	0.589 ± 0.034ab	*	*	ns
	18a	0.529 ± 0.045a	0.579 ± 0.043ab	0.603 ± 0.038ab			
	25a	0.535 ± 0.050a	0.564 ± 0.007ab	0.564 ± 0.003b			
	34a	0.588 ± 0.078a	0.583 ± 0.061ab	0.616 ± 0.001a			
	44a	0.582 ± 0.023a	0.618 ± 0.012a	0.630 ± 0.022a			
	Average	0.564B	0.576AB	0.600A			

Data were shown as means values ± S.E. (*n* = 3 or 15). Different uppercase and lowercase letters indicate significant differences among different soil aggregates and differently-aged *Larix principis-rupprechtii* stands within the same soil aggregates at the *P* < 0.05 level, respectively. Two-way ANOVA are applied for identifying the effects of stand age (S), aggregate size (A), and their interaction (S × A) on the above-mentioned indices (The results are shown in the right side of the table). **, *, and “ns” represent significant at *P* < 0.01, *P <* 0.05, and no significant differences (*P* > 0.05), respectively. RI, recalcitrance index; AI, aromaticity index; A/OA, alkyl C/O-alkyl C.

### 3.7 Interaction mechanisms between microbial attributes and SOC characteristics

**Figure 5:**
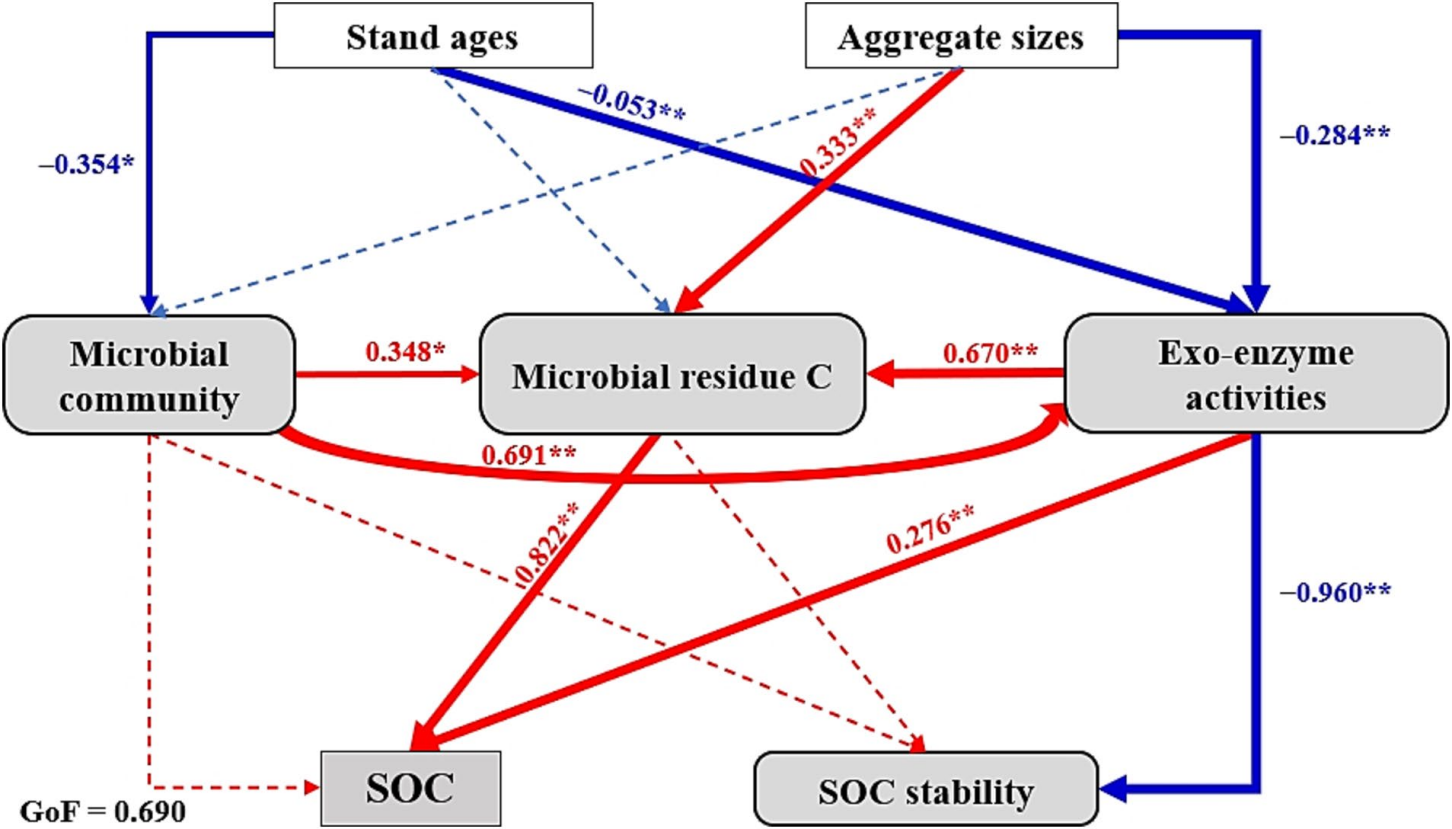
Partial least squares path model (PLS-PM) disentangling major pathways of microbial characteristics (i.e., microbial community, exo-enzyme activities, and microbial residue C) influences on SOC contents and their stability within aggregates across differently-aged *Larix principis-rupprechtii* stands. The arrows indicate the hypothesized direction of causation. The solid red and blue arrows indicate significant (*p* < 0.05) positive and negative relationships, respectively. Besides, the dashed arrows indicate non-significant effect (*p* > 0.05). The following variables were included: microbial community (total PLFAs, fungi, bacteria, and actinomycetes), exo-enzyme activities (*α*-Glucosidase, *β*-Glucosidase, β-Cellobiosidase, β-Xylosidase, N-acetyl-glucosaminidase, phenol oxidase, and peroxidase) and SOC stability (recalcitrance index, aromaticity index, and alkyl C/O-alkyl C). Asterisks indicate the statistical significance (^**^p < 0.01 and ^*^p < 0.05).

The partial least squares path modeling (PLS-PM, GOF = 0.690; Figure 5) was conducted to investigate the correlation between microbial attributes (i.e., microbial community, exo-enzyme activities, and microbial residues) and SOC characteristics within soil aggregates of different-aged larch plantations and their potential mechanisms. The PLS-PM analysis validated the hypothesis (iii) and demonstrated that stand ages have significant and negative impacts on microbial communities (path coefficient = −0.354^**^) and exo-enzyme activities (−0.053^**^); concurrently, the aggregate sizes exhibited significantly positive and negative effects on microbial residue C (0.333^**^) and exo-enzyme activities (−0.284^**^), respectively. Besides, microbial community (0.348^*^) and exo-enzyme activities (0.670^**^) together positively regulated microbial residue C. Furthermore, microbial residue C (0.822^**^) and exo-enzyme activities (0.276^**^), rather than microbial community, were considered to be critical factors that positively affected SOC contents. Simultaneously, exo-enzyme activities (−0.960^**^) exhibited a significantly negative effect on SOC stability.

## 4 Discussion

### 4.1 Changes in soil aggregate distribution and nutrient characteristics along stand development

Soil aggregate’s characteristics (i.e., aggregate distribution, MWD, and GMD), essential indicators for evaluating soil structure and quality, are governed by a series of factors, e.g., organic matter, Fe/Al oxides, and other biological attributes (Cheng et al., 2024). In this study, the proportion of > 2 mm aggregates and aggregate stability (as indicated by the values of MWD and GMD) increased along the stand ages, reaching the highest in the 18a stand (Table 1). This finding was consistent with previous study at the Beijiang River Forest Farm in Guangxi Zhuang Autonomous Region, China (He et al., 2021), which suggested with increasing stand ages, the proportions of > 2 mm aggregates and MWD initially increase and then decrease, and peak in the 17a stand. These findings could be ascribed to the temporal changes in organic C contents in soils during forest development (Ayoubi et al., 2012). Sun et al. (2024) also revealed that organic C in soils served as key “binding agents” for aggregate formation and enhancing aggregate stability. The similar temporal variation patterns between organic C and MWD in the present study also confirmed the above-mentioned opinions.

The stocks of SOC and nutrients (e.g., nitrogen) depends on the dynamic balance of exo-resources inputs (e.g., litters) vs. outputs (e.g., mineralization) (Luan et al., 2022). Generally, it is expected that SOC and nutrients would increase with stand age due to the annual input and accumulation of forest litters and root exudates in the subtropical regions in China (Chen et al., 2020; Hu J. et al., 2024). Nevertheless, our findings indicated that the contents of SOC and TN within aggregates initially increase, then decrease, peaking in the 18a stand (Table 2), which were contradicted the hypothesis (i). These inconsistent findings may be explained by the differences in climatic conditions across different study regions (i.e., subtropical region vs. temperate region in this study). Ma et al. (2022) highlighted that temperature, as one vital part of soil microclimates, may alter microbial growth and functional characteristics, thereby indirectly affecting litter degradation and SOC cycling processes. Donhauser and Frey (2018) also demonstrated that several adverse soil microenvironment, e.g., severely low temperature and desiccation, can impose significant stress on microbial communities, potentially inhibiting their activity and growth. Consequently, the climatic conditions characterized by low temperature (annual average temperature of −1.4°C) at the study sites impede microbial activity and litter decomposition (Tian et al., 2025). Meanwhile, the annual storage of litter detritus led to the development of thicker litter layers in the elder stands, which diminished soil aeration, thereby further inhibiting litter decomposition (Keiluweit et al., 2016). In other words, the continuous cold temperature and progressively worsening aeration conditions in soils were not beneficial for the conversion of litter into SOC, resulting in a reduction of SOC in the 25a, 34a and 44a stands in this study.

Yao Y. et al. (2019) demonstrated that SOC and nutrients were unevenly distributed across aggregates. In the present study, the contents of SOC and TN showed a similar distribution pattern within different aggregates, i.e., higher contents of SOC and TN were basically observed in the larger aggregates than those in the smaller aggregates irrespective of the stand ages (Table 2), which were verified the hypothesis (ii) and indicated that larger aggregates owned a higher capacity to retain these nutrients. This phenomenon could be explained by the “aggregate hierarchy model,” which posits that larger aggregates are constituted by smaller aggregates in conjunction with several “binding agents” (e.g., organic C constitutes); meanwhile, this process facilitated the storage of C within the larger aggregates (Ji et al., 2024). Wang et al. (2017) also reported that exogenous C and nutrient (e.g., forest litters) resources are difficult to reach the smaller aggregates due to the preferential “localization” of these resources within the larger aggregates, which further confirmed that the larger aggregates contained higher SOC and TN than the smaller aggregates in the present study.

### 4.2 Changes in microbial attributes within aggregates along stand development

**Figure 6:**
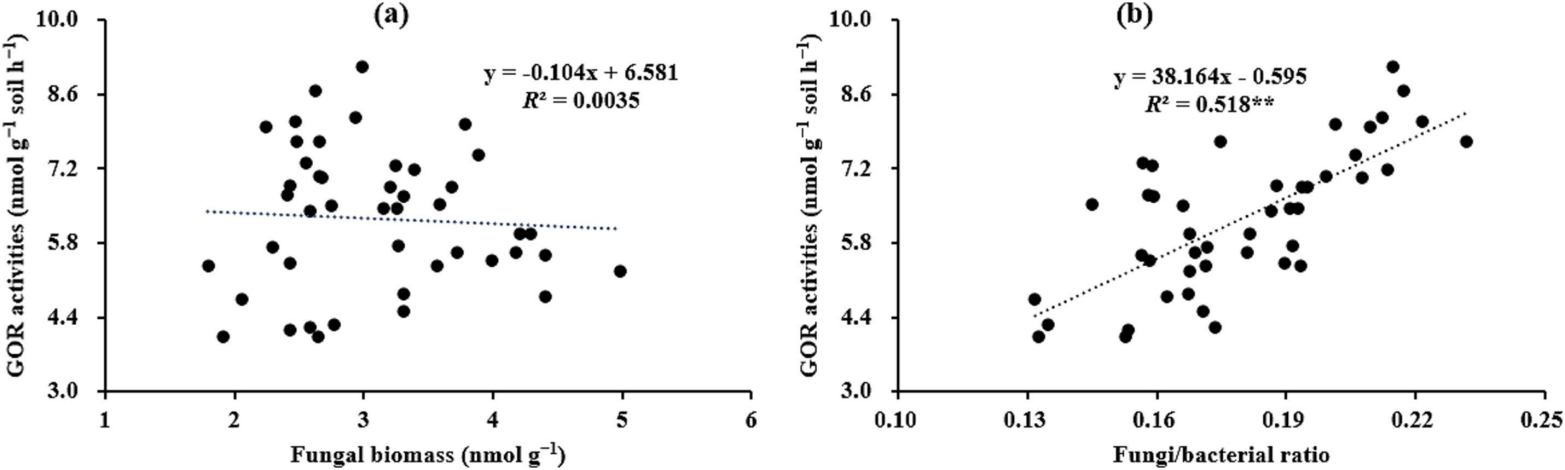
Relationships between the GOR activities and the fungal biomass **(a)** or the fungi/bacteria ratio **(b)** within soil aggregates across differently-aged *Larix principis-rupprechtii* stands (*n* = 45). GOR, the geometric mean of the oxidative exo-enzyme activities; F, fungi; B, bacteria. ^**^Indicates significant at *p* < 0.01.

Given the important role of microbes in the process of SOC cycling, clarifying the temporal–spatial changes in microbial attributes (e.g., microbial community, function, and metabolism) can provide valuable information for better understanding the mechanisms of SOC stabilization during forest development (Zhang et al., 2021). In the present study, it was observed that as stand age increased, microbial biomass (as indicated by the contents of total PLFAs; Table 3), hydrolytic exo-enzyme activities (Figure 3), and microbial residue C (Figure 4) within aggregates, initially increased and then decreased, and the highest values for these indices were observed in the 18a or 25a stands. This was partially aligned with the findings of Zhao et al. (2019), who reported microbial biomass showed a unimodal pattern with the progression of afforestation through meta-analysis. The likely reasons for these findings are as follows. Francini et al. (2018) revealed that appropriate soil microenvironment (e.g., adequate nutrients and O_2_) is the prerequisites for facilitating microbial growth and metabolism. In the present study, the better soil nutrient status (i.e., higher TN level) in the 18a and 25a stands, as presented in Table 2, created favorable soil conditions for microbial growth and metabolism, which caused higher contents of microbial biomass and microbial residue C in these stands. Sokol et al. (2022) indicated that the exo-enzymes, i.e., hydrolytic and oxidative exo-enzymes, are mainly secreted and produced by bacteria and fungi, respectively, with their activities being mainly regulated by soil properties (e.g., pH) and microbial community structure, etc. Therefore, the higher microbial biomass (Table 3) in the 18a and 25a stands were conducive to enhance hydrolytic exo-enzyme activities. Interestingly, we found that the variations in oxidative exo-enzymes, i.e., the gradually increasing oxidative exo-enzyme activities (as indicated by the values of GOR) during forest development (Figure 3b), were positively correlated with the values of F/B (Figure 6b), rather than with the contents of fungi (Figure 6a). Wang and Kuzyakov (2024) demonstrated that fungi can enhance their competitive ability for nutrients against bacteria by secreting more oxidative exo-enzymes under the premise of limited soil nutrient resources. Namely, although the restricted soil nutrient resources in the elder stands was not favorable for microbial growth (Table 3), they can stimulate fungi to secrete more oxidases to cope with this situation, which partially corroborates the results of Figure 6 and accounts for the observed variations in GOR across differently-aged stands in the present study.

Nowadays, the distribution patterns of microbial attributes within aggregates have been widely reported, but these results remain controversial (Wang S. et al., 2018; Murugan et al., 2019). Our findings indicated that larger aggregates owned higher microbial biomass (Table 3) and hydrolytic exo-enzyme activities, alongside reduced oxidative exo-enzyme activities (Figure 3), compared to smaller aggregates, regardless of the stand ages. This could be explained by exo-enzyme function and aggregate’s characteristics (Yao S. et al., 2019; Sokol et al., 2022). The higher nutrient level (see Table 2) as well as more pore structure in the larger aggregates (Wang and Hu, 2023), rather than smaller aggregates, could create a better microenvironment for microbial growth. Furthermore, the nutrient deficiency in the smaller aggregates (see Table 2) may exacerbate bacterial–fungal competition for soil resources, thereby causing fungi to secrete more oxidative exo-enzymes to meet this competition (Wang and Kuzyakov, 2024). Notably, the increased FRC/BRC ratios observed in the smaller aggregates (Figure 4) suggested that FRC played a more dominant role than BRC in forming SOC in these aggregates. The possible explanation for these findings could be associated with differences in microbial necromass properties (Ding et al., 2010) and microbial community structure among different aggregates (Wang S. et al., 2018). He et al. (2022) indicated that fungal and bacterial biomass are the prerequisites for the formation of FRC and BRC, respectively. Hu J. et al. (2024) demonstrated BRC is relatively unstable and more susceptible to decomposition compared to FRC in soils characterized by “poor” nutrient levels. Based on the above-mentioned findings, the lower SOC and TN, along with higher F/B values observed in the smaller aggregates (Tables 5, 6), led to increased consumption of BRC and enhanced production of FRC, which explained the higher FRC/BRC in the smaller aggregates.

### 4.3 Changes in SOC characteristics within aggregates along stand development

Understanding the temporal dynamics in SOC chemical composition within aggregates can provide several new insights for better clarifying the mechanisms of SOC stabilization process during forest development (Wu et al., 2021). In the present study, the lower O-alkyl C abundance and higher aromatic C abundance were observed in the older stands (34a and 44a) compared to the younger stands (Table 6). This result was consistent with observations from Yang et al. (2021), in which the 64a stands contained higher aromatic C abundance and lower O-alkyl C abundance than the 19a and 37a stands in the Moso bamboo plantations. This could be mostly ascribed to the imbalance between soil resource vs. microbial nutrient demand (Gurmessa et al., 2024). The O-alkyl C (i.e., the labile C constitutes), rather than aromatic C (i.e., the stable C fractions), were preferentially consumed by microbes for their growth and metabolism under the “poor” soil nutrient status in the older stands (Table 2) (Yao S. et al., 2019). Notably, in accordance with previous studies (Luan et al., 2021; Wang et al., 2022), we observed that the SOC in the larger aggregates owned lower O-alkyl C abundance and higher aromatic C abundance than those in the smaller aggregates. The reduced nutrient levels observed in the smaller aggregates (see Table 2) could enhance the consumption of labile C (e.g., O-alkyl C) by microbes (Gurmessa et al., 2024), which could partially elucidate the above-mentioned findings. Besides, fungi are recognized as the key decomposers of stable C constitutes (e.g., aromatic C) and preferentially thrive in soils with high O_2_ levels (Liu et al., 2021). Consequently, the diminished fungal biomass and poor gas (e.g., O_2_) status in the smaller aggregates are not favorable for the decomposition of recalcitrant C (e.g., aromatic C). Taken together, the above-mentioned findings offer an explanation for the variations in SOC functional group composition across different aggregates.

The RI, AI, and A/OA were calculated by the four C functional groups, which could be applied to evaluate SOC stability, aromaticity, and degradation degree, respectively (Guo et al., 2019). In this study, as aggregate sizes decreased, these indices (i.e., RI, AI, and A/OA) showed increasing trends (Table 6), indicating that SOC in the smaller aggregates exhibited higher stability, greater aromaticity, and a higher degree of degradation. These rising patterns with decreasing aggregate sizes are likely attributed to enhanced consumption of O-alkyl C and diminished decomposition of aromatic C in the smaller aggregates (Table 6; Guo et al., 2019). The above-mentioned findings, in conjunction with our results (i.e., elevated RI, AI, and A/OA values in the 44a stand), also suggested an enhancement in SOC stability and aromaticity in the older stands.

## 5 Conclusion

The elucidation of the temporal dynamics of SOC characteristics and microbial attributes at the aggregate scale, employing a series of technologies (i.e., ^13^C NMR, PLFAs, exo-enzymatic activity, and microbial residue analysis, etc.), can offer novel insights into SOC stocks in relation to stand ages in *Larix principis-rupprechtii* plantations in Northern China. Firstly, with increasing stand age, the levels of SOC, microbial biomass, hydrolytic exo-enzymatic activities, and microbial residues, initially increase and then decrease, and generally peak in the 18a stand; meanwhile, these indices were generally higher in larger aggregates compared to smaller aggregates irrespective of the stand ages. Secondly, we observed that the increased stand ages and reduced aggregate size altered SOC chemical composition (more “labile” O-alkyl C and less “stable” aromatic C), enhanced SOC stability and oxidative exo-enzymatic activities, as well as enlarged the microbial contribution to SOC stocks. Finally, the PLS-PM model confirmed that SOC stocks were closely linked to microbial residues; meanwhile, the microbial community altered SOC stability by modulating exo-enzyme activities. These findings enhance our understanding of the microbial-driven mechanisms underlying SOC stabilization during forest development in Northern China.

## Data Availability

The data analyzed in this study is subject to the following licenses/restrictions: requests to access these datasets should be directed to luanhaoan@163.com.
