# Supplementary material for: Linkage of living microbial biomass, function, and necromass to soil organic carbon storage along a chronosequence of Larix principis-rupprechtii plantation in North China
Source: Front Microbiol. 2025 May 26;16:1588030. doi: 10.3389/fmicb.2025.1588030 (PMC12146326; doi:10.3389/fmicb.2025.1588030)
Supplement: Supplementary file 1 [file Table_1.docx]

Table S1 The microbial subgroups indicated by fatty acid biomarker groups in the present study (Peng et al., 2016).

| Microbial subgroups | Specific fatty acids |
| --- | --- |
| Bacteria | a13:0, i13:0, a14:0, i14:0, a15:0, i15:0, a16:0, i16:0, a17:0, i17:0, a19:0, 15:1ω5c, 16:1ω7c, 16:1ω7c DMA, cy17:0, 10Me-17:1ω7c, 17:1ω8c, 18:1ω5c, 18:1ω7c, 10Me-18:1ω7c, cy19:0, 15:00, and 17:00 |
| Fungi | 16:1ω5c, 18:2ω6c, and 18:1ω9c |
| Actinomycetes | 10Me-16:0, 10Me-17:0, and 10Me-18:0 |
| Other microbes | 14:00, 16:00 and 18:00 |

^#^ The sum of bacteria, fungi, actinomycetes, and other microbes was expressed as the total microbial biomass.

Table S2 A detailed description of exo-enzymes in the present study (Zhang et al., 2015).

| Soil exo-enzymes | | Substrate | Abbreviation |
| --- | --- | --- | --- |
| Hydrolytic exo-enzymes | α-Glucosidase | 4-MUB-α-D-glucoside | αG |
|  | β-Glucosidase | 4-MUB-β-D-glucoside | βG |
|  | β-Cellobiosidase | 4-MUB-β-D-cellobioside | CBH |
|  | β-Xylosidase | 4-MUB-β-D-xyloside | XYL |
|  | N-acetyl-glucosaminidase | 4-MUB-N-acetyl-β-D-glucosaminide | NAG |
| Oxidative exo-enzymes | Phenol oxidase | *L*-DOPA | PHOs |
|  | Peroxidase | *L*-DOPA | PerX |
